# Supplementary material for: The oral pathogen Porphyromonas gingivalis gains tolerance to the antimicrobial peptide DGL13K by synonymous mutations in hagA
Source: PLoS One. 2024 Oct 24;19(10):e0312200. doi: 10.1371/journal.pone.0312200 (PMC11500903; doi:10.1371/journal.pone.0312200)

Fig S1. Raw gel image for Fig 2.

The original gel image contains three sets (A, B,C) of 10 lanes. Lanes marked with X are not included in Fig 2. Lane sets B and C are not included in Fig 2. The image was captured on a BioRad gel imaging system.

Lane A1: M_r markers

Lane A2: WT - 16S RNA

Lane A3: WT - *hag*

Lane A7: *hagA23-176* – 16S RNA

Lane A8: *hagA23-176 - hagA*

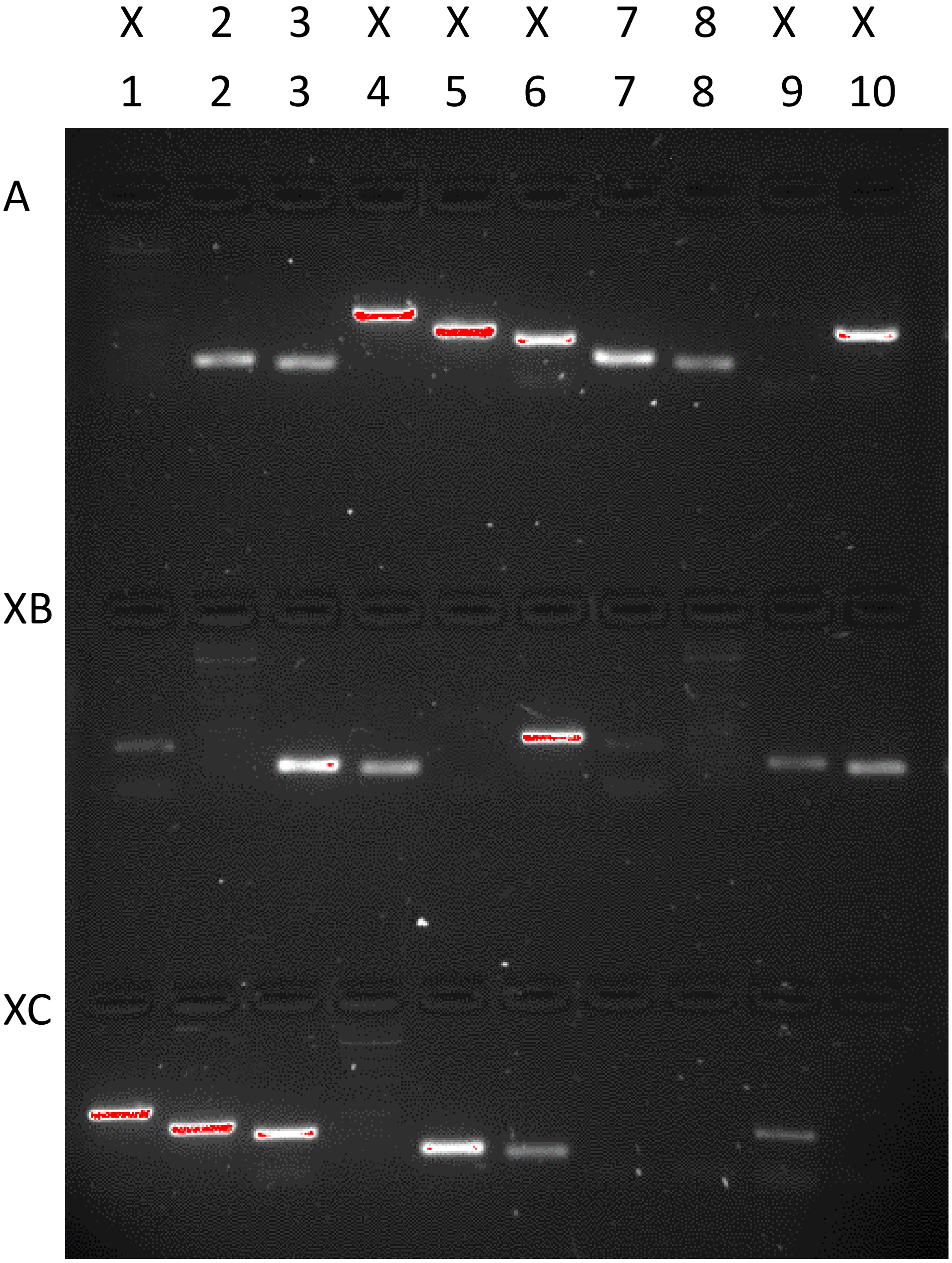

Supplement: S1 Fig — The original gel contains three sets (A, B,C) of 10 lanes. Lanes marked with X are not included in Fig 2. Lane sets B and C are not included in Fig 2. Lane A1: Mr markers; Lane A2: WT - 16S RNA; Lane A3: WT–hagA; Lane A7: hagA23-176 – 16S RNA; Lane A8: hagA23-176 –hagA. The image was captured on a BioRad gel imaging system. (PDF) [file pone.0312200.s001.pdf]
