# Supplementary material for: The oral pathogen Porphyromonas gingivalis gains tolerance to the antimicrobial peptide DGL13K by synonymous mutations in hagA
Source: PLoS One. 2024 Oct 24;19(10):e0312200. doi: 10.1371/journal.pone.0312200 (PMC11500903; doi:10.1371/journal.pone.0312200)
Supplement: S2 Fig — Supplement to the predicted structures shown in Fig 3, obtained by modeling the secondary structures by predicting the minimum free energy (MFE). The predicted structures show a 493 nucleotide segment of the mRNA sequence coding for the C-terminal K3 cleaved adhesin domain of hagA. (PDF) [file pone.0312200.s002.pdf]

Fig S2. MFE predicted RNA structure. Supplement to the predicted structures shown in Fig 3, obtained by modeling the secondary structures by predicting the minimum free energy (MFE). The predicted structures show a 493 nucleotide segment of the mRNA sequence coding for the C-terminal K3 cleaved adhesin domain of *hagA*.

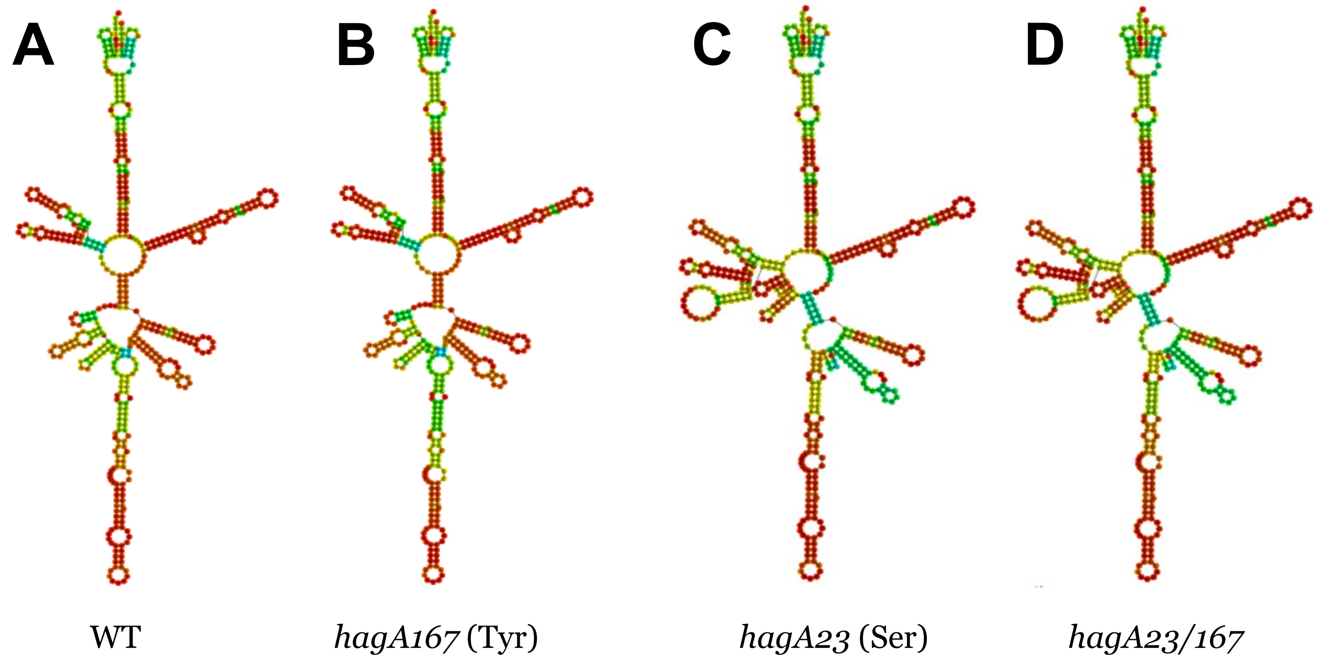

A. Model of WT

B. Model of a hypothetical *hagA167* mutant, which was altered at the Tyr codon at position 1936167.

C. Model of a hypothetical *hagA23* mutant, which was altered at the Ser codon at position 1936023.

D. Model of *hagA23/167*.
